# Individuals with Diabetes Mellitus Have a Dry Eye Phenotype Driven by Low Symptom Burden and Anatomic Abnormalities

**DOI:** 10.3390/jcm12206535

**Published:** 2023-10-15

**Authors:** Elyana V. T. Locatelli, Jaxon J. Huang, Simran Mangwani-Mordani, Arianna A. Tovar Vetencourt, Anat Galor

**Affiliations:** 1 Surgical and Research Services, Miami Veterans Affairs Medical Center, Miami, FL 33125, USA; elocatelli2017@fau.edu (E.V.T.L.); jaxonjh@hawaii.edu (J.J.H.); simranmangwanim@gmail.com (S.M.-M.); 2 Bascom Palmer Eye Institute, University of Miami, Miami, FL 33136, USA; atovarvetencourt@augusta.edu

**Keywords:** diabetes mellitus, eyelid laxity, dry eye disease, floppy eyelids, anterior segment

## Abstract

Dry eye disease is an umbrella term that includes a variety of symptoms and signs. A link between diabetes mellitus and dry eye disease exists, but the associated phenotype needs further examination. Thus, our aim was to determine how diabetes mellitus relates to the dry eye disease phenotype. A prospective, cross-sectional study was conducted at the Miami Veteran Affairs Medical Center ophthalmology clinic between October 2013 and September 2019. Participants included a volunteer sample of 366 South Florida veterans with one or more symptoms or signs of dry eye disease [Dry Eye Questionnaire-5 ≥ 6 OR tear break-up time ≤ 5 OR Schirmer’s test score ≤ 5 OR corneal fluorescein staining ≥ 2]. Participants were divided into three groups: (1) individuals without diabetes mellitus (controls); (2) individuals with diabetes mellitus but without end-organ complications; and (3) individuals with diabetes mellitus and end-organ complications. Dry eye metrics were compared across groups. The main outcome measures included ocular symptom questionnaires [e.g., 5-item Dry Eye Questionnaire, Ocular Surface Disease Index, and ocular pain assessment] and clinical parameters obtained from an ocular surface evaluation. A total of 366 individuals were included (mean age 59 ± 6 years; 89% males; 39% White; 11% diabetes mellitus and end-organ complications; 15% diabetes mellitus but without end-organ complications). Individuals with diabetes mellitus and end-organ complications had lower symptom scores on the dry eye disease and pain-specific questionnaires compared to individuals with diabetes mellitus but without end-organ complications and controls (Ocular Surface Disease Index: 42.1 ± 24.5 vs. 38.9 ± 25.1 vs. 23.6 ± 16.2; *p* < 0.001; numerical rating scale of ocular pain intensity: 4.9 ± 3.2 vs. 4.3 ± 2.7 vs. 3.5 ± 2.7; *p* = 0.02). Eyelid laxity was also more severe in the group with diabetes mellitus and end-organ complications (0.69 ± 0.64 vs. 0.73 ± 0.72 vs. 1.08 ± 0.77; *p* = 0.004) compared to the two other groups. The diabetic dry eye disease phenotype is driven by signs more so than by symptoms, with anatomic eyelid abnormalities being more frequent in individuals with diabetes mellitus and end-organ complications. Given this, ocular surface abnormalities in individuals with DM may be missed if screened by symptoms alone. As such, individuals with DM should undergo a slit lamp examination for signs of ocular surface disease, including anatomic abnormalities.

## 1. Introduction

Dry eye disease (DED) is a prevalent, multifactorial disease encompassing a broad range of ocular symptoms and clinical presentations [1]. The key factors implicated in DED pathophysiology include ocular surface inflammation, tear film instability, hyperosmolarity, and neurosensory abnormalities [2]. However, other factors such as anatomic abnormalities (e.g., floppy eyelid syndrome and conjunctivochalasis) and meibomian gland dysfunction (MGD) often coexist with primary tear deficiencies, adding to the many presentations of DED [3]. The current stratification method for DED relies on two major forms, aqueous tear deficiency (ATD) and evaporative deficiency, but there is an overlap in symptoms and signs between groups [4]. Moreover, patient-reported symptoms and clinical signs of this disease are often discordant, with some individuals having symptoms out of proportion to its signs and others having ocular surface abnormalities with minimal symptoms, thus adding to the challenge in evaluation [4]. Regardless of etiology or presentation, DED can be chronic and debilitating, affecting a patient’s quality of life and mental health [3,5].

In 2017, the Dry Eye Workshop II Reports published a summary of the risk factors for DED based on consistent or probable findings within the literature, some of which include demographics (e.g., female sex and older age), oral medication use (e.g., antidepressants and anxiolytics), hormone abnormalities (e.g., androgen deficiency), and immunologic (Sjogren’s syndrome and systemic lupus erythematosus) and metabolic (e.g., diabetes) diseases [6,7]. The field is limited, however, in its understanding regarding how specific risk factors relate to specific phenotypes of DED. Many studies have relied upon the International Classification of Disease (ICD) codes or symptoms alone for DED identification, a method lacking the information necessary to discern which DED subtypes most closely align with a particular factor [7,8]. Thus, it is also unclear which interventions and treatment strategies are most effective for different etiologies of DED. As an important first step in bridging the gap in the literature between specific comorbidities and DED phenotypes, our study looks at diabetes mellitus (DM), given that patients with DM constitute a high-risk population for DED [8].

DM is a major public health problem and one of the most prevalent systemic diseases in the world, with a globally increasing prevalence noted over the past three decades [9]. DM-associated ophthalmological complications are common and are a leading cause of blindness worldwide [10]. Some DM-related ocular complications, such as diabetic retinopathy (DR), are well studied, but others, particularly those involving the anterior segment, are not as well described. However, studies that are available indicate associations between DM and DED, including a marked increase in the prevalence of DED in individuals with DM compared to non-diabetic controls [8,10,11,12,13,14]. For example, a case–control study of individuals with and without DM (n = 120 in each group) reported a significant difference between groups (38.3% vs. 25.0%; *p* = 0.02, respectively) in the prevalence of one sub-type of DED (Schirmer’s test score ≤ 10 mm) [14].

Despite several reported associations between DM and DED, the DED profiles, symptomatically and clinically, are largely varied among DM individuals, and thus, the underlying mechanism through which this occurs remains undistinguished [15]. For example, scores on the Ocular Surface Disease Index (OSDI) questionnaire, which assesses DED symptoms according to severity, triggers, and effect on vision-related function [16], lack consistency across populations. In one study, individuals with DM, both type 1 (DM1) and type 2 (DM2), had higher OSDI scores compared to controls without DM (DM1: 26 ± 16; DM2: 33 ± 23; controls: 21 ± 8; *p* < 0.05 for both comparisons) [13]. In contrast, a study that categorized DM and control participants (n = 120 in each group) based on OSDI score ranges (normal: 0–12, mild: 13–22, moderate: 23–32, and severe: >32 points) did not find significant differences in OSDI severity between the groups [14].

Variability has similarly been noted with regard to DED signs. For example, one study found decreased tear stability (measured based on tear break-up time, TBUT) (8.25 ± 0.25 vs. 13.22 ± 1.87 s; *p* < 0.001) and tear production (Schirmer’s test scores: 8.75 ± 2.24 vs. 16.67 ± 2.41 mm wetting; *p* < 0.001) in individuals with DM compared to controls [11]. However, other studies did not duplicate these findings and found similar tear stability (3.79 ± 2.25 vs. 3.99 ± 2.60 s; *p* = 0.49) and tear production (Schirmer’s test scores: 5.57 ± 4.70 vs. 6.55 ± 5.93 mm; *p* = 0.43) in DM patients versus controls [17]. Similar inconsistencies are present with regard to MGD and corneal neurosensory features by DM status [18]. The evolving understanding that different comorbidities align with different DED subtypes [1], combined with the complex pathophysiology of DM, prompts a more in-depth study on the link between DM and DED. A better understanding of DM-related DED can allow for more targeted approaches that will hopefully translate to improved function and quality of life.

## 2. Materials and Methods

Study population: Individuals between 40 and 70 years of age with DED symptoms or signs were recruited prospectively from the Miami Veterans Affairs (VA) Healthcare System eye clinic between October 2013 and September 2019. Individuals met the inclusion criteria for DED by having one or more of the following: 5-item Dry Eye Questionnaire (DEQ-5) score ≥ 6, TBUT ≤ 5 s, anesthetized Schirmer’s test score ≤ 5 mm of wetting at 5 min, or corneal fluorescein staining (CFS) score ≥ 2, in either eye. The exclusion criteria included subjects with an active external process; overt corneal (e.g., keratoconus), conjunctival (e.g., pterygium), or eyelid (e.g., ectropion) abnormalities; cataract surgery within the last 6 months; a history of any refractive, glaucoma, or retinal surgery; contact lens wear; ocular medications beyond artificial tears; or a diagnosis of Sjögren’s syndrome, collagen vascular disease, sarcoidosis, or graft-versus-host disease. Informed consent was obtained from all subjects. Approval from the Miami VA Institutional Review Board was obtained to allow prospective evaluation of the included subjects. This study was conducted in accordance with the principles of the Declaration of Helsinki and complied with the requirements of the United States Health Insurance Portability and Accountability Act.

Demographics and clinical data: Demographics, including age, sex, race and ethnicity, and medical history, were obtained via self-report and verified through chart review. The pertinent clinical information that was collected included the most recent HbA1C value (glycosylated hemoglobin), weight, and height, if available. Body mass index (BMI) was calculated by dividing weight (in pounds) by height (in inches) squared and multiplying by 703 to obtain the units in kilograms/meters^2^ (kg/m^2^).

DM group classifications: Our DM sample constitutes both types of DM, including individuals with DM1 or DM2. For individuals with a diagnosis of DM, additional information was collected, including history of DM-related complications, such as retinopathy, nephropathy, neuropathy, and cardiovascular, peripheral vascular, and cerebrovascular diseases. Retinopathy was considered present if specified as diabetic retinopathy (presence of retinal hemorrhages, exudates, cotton-wool spots, or neovascularization) or diabetic macular edema. Nephropathy was considered present if there were early signs (positive urine albuminuria, either spot urine with albumin-to-creatinine ratio ≥ 3.39 mg/mmol or a positive urine dipstick) or late signs (diagnosis of chronic kidney disease). Neuropathy was considered present if specified as diabetic peripheral neuropathy, as evidenced by the presence of paresthesias and reduced sensory and vibration perception. Cardiovascular disease was considered present if the subjects had any of the following diagnoses: coronary artery disease, cardiomyopathy, or congestive heart failure. Peripheral vascular disease included peripheral arterial disease and foot ulcers, and cerebrovascular disease included strokes and transient ischemic attacks. Participants were divided into three groups based on their DM status: (1) individuals without DM (controls); (2) individuals with DM but without end-organ complications (DM − C); and (3) individuals with DM and 1 or more of the above end-organ complications (DM + C). 

Questionnaires: All subjects completed a medical history form which included demographics, past ocular and medical history, and medication information. Participants completed standardized questionnaires regarding DED symptoms (DEQ-5: 0–22 [19] and OSDI: 0–100 [16]) and ocular pain symptoms (numerical rating scale (NRS): scores 0–10 for “average intensity of eye pain during the past week” [20]) and selected questions from the Neuropathic Pain Symptoms Inventory Modified for the Eye (NPSI-Eye). The range of scores for the selected questions from the NPSI-Eye questionnaire was 0–40 (0 to 10 for individual questions, with 4 in total), and these questions assessed neuropathic ocular pain symptoms based on response to stimuli presented on or to the eye (i.e., burning, wind, light, and contact with a hot or cold stimulus) [21]. 

Corneal sensitivity: Mechanical detection and pain thresholds of the central cornea were assessed using a modified Belmonte non-contact aesthesiometer [22]. The tip of the aesthesiometer (0.5 mm in diameter) was placed perpendicular to, and 4 mm from, the surface of the cornea of the right eye. The stimulation consisted of air pulses at room temperature (~23 to 26 °C) applied to the corneal surface. The method of limits, using ascending series only, was used to measure the detection thresholds. For the test, the subjects were presented with a stimulus immediately following a blink and asked to indicate whether they felt the stimulus by pressing a button. The initial flow rate was set at a level below the threshold (50 mL/min for most individuals) and increased by 10 mL/min (with 15 s intervals between stimuli) until the subjects stated that they felt the stimulus or the maximum allowable flow rate (400 mL/min) was reached. Two ascending series were conducted, and the detection threshold was defined as the arithmetic mean of the value at which the subjects pressed the button across the two series. 

Ocular surface health: All subjects underwent a standard tear film assessment, including measurement of the following (in the order assessed):

(1) Tear film osmolarity (TearLAB, San Diego, CA, USA) [23];
(2) Assessment of upper or lower eyelid laxity as determined based on rotation (0 = 0–25%; 1 = 25–50%; 2 = 50–100%) and the snapback test (0 = prompt snapback; 1 = slow return; 2 = does not return fully until blinking) [24], respectively;
(3) Inferior meibomian gland plugging graded on a scale from 0 to 3 (0 = none; 1 = less than 1/3; 2 = between 1/3 and 2/3; 3 = greater than 2/3 lid involvement, graded without contact) [25];
(4) Telangiectasias of the lower eyelids (0 = none; 1 = mild; 2 = moderate; 3 = severe) [25];
(5) Assessment of conjunctivochalasis (0 = absent vs. 1 = present) in each area of the lower eyelid (nasally, medially, and temporally);
(6) Non-invasive tear film stability based on TBUT (5 µL of fluorescein placed, 3 measurements taken in each eye and averaged) [26];
(7) Fluorescein corneal staining graded according to the National Eye Institute (NEI) scale [26], with 5 areas of the cornea being assessed, including the inferior, nasal, superior, temporal, and central areas, and scored from 0 to 3 in each (max. total = 15);
(8) Tear production graded based on mm wetting of anesthetized Schirmer’s test placed in the inferior fornix for 5 min (300 s) [26];
(9) Meibum quality graded based on a scale from 0 to 4 (0 = clear; 1 = cloudy; 2 = granular; 3 = toothpaste; 4 = no meibum extracted) [25];
(10) Inferior eyelid meibomian gland dropout graded according to the Meiboscale (0 = no dropout; 1 ≤ 25% dropout; 3 = 25% to 75% dropout; 3 ≥ 75% dropout) [27].

Statistical Analysis: Statistical analyses were performed using the SPSS 28.0 (SPSS Inc, Chicago, IL, USA) statistical package. Descriptive statistics presented as mean ± standard deviation (SD) or percentages were used to summarize patient and clinical information. Analysis of variance (ANOVA) and post hoc least significant difference (LSD) test were used to examine the mean differences between the groups. Multivariate linear regression analyses were used to further analyze the relationships among the variables. 

## 3. Results

### 3.1. Study Sample

The study sample consisted of 366 South Florida veterans with symptoms (DEQ-5 ≥ 6: 92.3%, n = 338) or signs (TBUT ≤ 5 s: 14.8%, n = 54; Schirmer’s test ≤ 5 mm wetting: 15.0%, n = 55; or CFS ≥ 2: 41.8%, n = 153, in either eye) of DED, who met the inclusion and exclusion criteria. The mean age of the group was 59.2 ± 6.4 years; most individuals self-identified as male (89.3%), Black (59.6%), and non-Hispanic (75.4%). A minority of individuals had DM − C (14.5%) or DM + C (11.2%). Individuals with DM + C were older, were more likely to be male, had higher HbA1C values, and were less likely to take non-steroidal anti-inflammatory drugs (NSAIDs) than the other two groups (Table 1). 

### 3.2. DED Symptoms

As evidenced by the OSDI scores, participants with DM + C reported fewer DED symptoms than the other two groups. Participants with DM + C also had lower symptom scores than the controls on the pain-specific questionnaires (NRS and NPSI-Eye) (Table 2). 

### 3.3. DED Signs

There were no significant differences in corneal sensation, osmolarity, meibomian gland (MG) status (plugging, quality, and dropout), or tear parameters (TBUT, CFS, and Schirmer) across groups. However, participants with DM + C had higher eyelid vascularity scores and more abnormal anatomy (laxity and conjunctivochalasis) compared to participants with DM − C (Table 2). 

### 3.4. Multivariable Analyses

DED metrics that differed between the groups were examined further using multivariate forward stepwise linear regression analyses that included potential confounders, such as demographics (i.e., age), clinical data (BMI), co-morbidities (hypertension and hypercholesterolemia), and medication use (NSAIDs, ASA, and statins), along with DM status. When considering all variables, DM remained a predictor of OSDI scores and eyelid laxity (Table 3), but not ocular pain, conjunctivochalasis, or eyelid vascularity.

## 4. Discussion

Our study found that participants with DM, particularly those with end-organ complications (DM + C), reported fewer DED symptoms but had more abnormal ocular anatomy than participants without DM-related complications (DM − C) and controls. The strongest signal was with respect to OSDI scores and eyelid laxity, which remained significantly associated with DM when considering potential confounders. As in the case of our study, a discrepancy between symptoms and signs of DED has previously been described in individuals with DM. For example, one study that divided individuals with MGD (defined as the presence of abnormal secretions, ≥2 telangiectasias, or plugging of ≥2 gland orifices) by symptom severity found associations between DM and asymptomatic MGD (adjusted odds ratio (OR): 2.23, 1.30–3.84; *p* ≤ 0.05), but not symptomatic (adjusted OR: 1.54, 0.72–3.30; *p* > 0.05) MGD [28], demonstrating reduced DED symptoms in those with DM. The disconnect between symptoms and signs of DED in DM patients is also supported by other studies, including one that reported DM as an independent predictor for symptom/sign DED discordance [29]. 

The interplay between DM and DED is likely complex and involves multiple facets of DM (e.g., hyperglycemia and oxidative stress) that can impact the lacrimal glands (LGs), the cornea, and nerve health [8,30,31,32]. One study investigated the impact of hyperglycemia and its treatment on the LGs and tear health in rats. Overall, LG activity and function were negatively impacted by DM, with higher oxidative stress levels (measured as higher total peroxidase activity) and impaired function (measured as higher lactoperoxidase levels) in DM versus control rats. Importantly, insulin-treated rats had similar levels of LG total peroxidase activity compared to control (non-DM) animals, demonstrating the beneficial impact of treatment on LG health. Rats with DM also had more abnormal tear metrics, with lower tear production compared to treated and non-DM rats (Schirmer’s test values: 2.5 ± 1.1 vs. 5.3 ± 0.3 vs. 8.0 ± 0.6 mm; *p* = 0.03) [32]. Although the existing data demonstrate a direct association between DM and aqueous tear-deficient DED in animal models, our study found no significant differences in tear parameters (e.g., Schirmer’s test) between DM + C patients, DM − C patients, and controls, while others have reported similar findings in human populations [17]. This further supports the heterogeneity observed in human populations.

Ocular surface inflammation, driven by the formation of advanced glycation end product (AGE)-modified proteins, may also be a link between DM and DED [33]. AGEs are glycated chemical compounds formed under conditions of extended sugar exposure and, once formed, are nearly irreversible [34]. When AGEs bind to their receptor (RAGE), a cascade of intracellular inflammation occurs with activation of the JAK-STAT (Janus kinase/signal transducer and activator of transcription 3) pathway [35]. One study that analyzed basal tears of individuals with DM + C and controls found higher levels of AGE-modified proteins in DM individuals compared to controls (0.68 ± 0.44 vs. 0.36 ± 0.21 µg/mg; *p* < 0.05) [36]. These findings may have implications for DED. One study examined two DED murine models (mutant mice lacking meibomian glands (MGD model) and lacrimal gland excision (aqueous tear deficiency (ATD) model)) and found that both case groups had STAT3 elevations in the cornea and conjunctiva (MGD group by 1.2- and 3.3-fold and ATD group by 1.78- and 1.3-fold, respectively) compared to control mice [37]. Thus, higher levels of AGEs in tears and local activation of the AGE-RAGE axis leading to ocular surface inflammation may provide a link between DM and DED. However, inflammation may not translate to abnormal clinical parameters as in our study, where osmolarity, MG plugging, meibum quality, and meibomian gland dropout were not significantly different between DM + C patients, DM − C patients, and controls. 

DM may also impact corneal nerves and lead to corneal neuropathy, which can lead to tear abnormalities due to inappropriate sensing and response to afferent signals [1,8]. A study comparing DM to non-DM mice found a significant reduction in corneal nerve terminal density (5.57 ± 0.94 vs. 9.02 ± 1.14%; *p* = 0.001) and sub-basal nerve plexus density (22.08 ± 1.78 vs. 34.77 ± 4.45 mm/mm^2^; *p* = 0.002) in DM compared to non-DM mice [38]. This study has human correlates as decreased corneal nerve density [39] and corneal sensitivity [12], with concomitant signs of neurotrophic keratitis (e.g., corneal staining) [40], have been noted in some, but not all, DM studies. Our findings, in particular, showed no significant differences in corneal sensation between DM + C patients, DM − C patients, and controls. One potential explanation for the heterogeneity of phenotypes is that treatment of hyperglycemia can impact disease manifestations and underlying pathophysiological mechanisms [32]. These complexities must be considered when examining DM manifestations in humans, where all individuals are treated with a variety of agents, including both oral agents and insulin. 

Fewer studies have focused on anatomic abnormalities in DM. However, unique to our study is the noted association between DM and eyelid laxity. One study stratified participants by the presence (n = 98) or absence (n = 40) of eyelid laxity but, unlike our study, did not find a relationship between laxity and DM (35% vs. 43%; *p* = 0.39) [41]. While the underlying pathophysiology is not fully understood, several hypotheses exist, including Demodex as a potential contributor and obesity as a shared co-morbidity [42]. Individuals with DM have been found to have a greater frequency of Demodex infestation compared to controls, as noted in epilation and microscopy evaluation (34.1% vs. 13.4%; *p* < 0.001, n = 255) [43]. Demodex produces a biofilm on the eyelid margins that can induce an inflammatory response (via inflammatory virulence factors like exotoxins, cytolytic toxins, and super-antigens), which may, over time, damage eyelid structures and manifest as laxity [44]. Unfortunately, we did not document clinical signs of Demodex (i.e., cylindrical dandruff) in our study and, thus, cannot further comment on this hypothesis. With respect to obesity, in our study, individuals with DM weighed more than their non-DM counterparts, but neither weight nor BMI remained a significant factor in the multivariable models that examined the relationship between DM and eyelid laxity. Nevertheless, there may be biological plausibility supporting a link between DM and eyelid laxity.

Together, these studies highlight the heterogeneity of DED presentations in DM patients, suggesting that multiple subtypes of DM-related DED exist. Given the various subtypes of pathophysiology of DED that can occur in individuals with DM, it is not surprising that there is such variability in DED symptoms and signs reported in the available literature. It is also important to note that some patients with DM may experience elements of neurotrophic keratitis (NK). Further studies are needed to elucidate the relationship between NK and DED in individuals with DM to clarify whether DED occurs within NK, or vice versa, and when one entity begins within the other. However, this study highlights the importance of anterior segment evaluations in individuals with DM, given the variety of pathologies that can occur.

As with all studies, our findings must be considered bearing in mind the study limitations, which include a selective study sample of predominantly older males that is not fully representative of all individuals with DM. However, men are often understudied with respect to DED and, thus, an important population to characterize. Information on the type of diabetes in our study sample was not collected, and the sample had differences in co-morbidities and medication usage that could have impacted DED presentation. In addition, the sample sizes across groups differed, which may have affected our results. Furthermore, our study did not collect information on nerve anatomy, Demodex infestation, and other potentially important confounders (i.e., diet and environmental conditions) that could have impacted our findings. Finally, we did not systematically test for DM, DM-related complications, or the extent of the complications; thus, the medical history collected for the participants in our study was based solely on self-report and chart review. 

Despite these limitations, our study highlights a paucity of symptoms and the presence of anatomic abnormalities of the eyelid in our DM patient sample, with individuals with DM and related complications having the lowest symptom severity and the highest degree of anatomic abnormalities. Our findings highlight a need to comprehensively examine individuals with DM as DED signs may be missed if only symptoms are used as a screening tool. An evolving area of interest is the exploration of therapies that may impact DED in individuals with DM, including diquafosol, topical naltrexone, and sodium/glucose cotransporter 2 (SGLT2) inhibitors [45,46,47]. Additionally, beyond tear parameters, an examination of anatomy and eyelid health is warranted in individuals with DM. When identified, these abnormalities may benefit from specific therapies (i.e., conjunctivoplasty or eyelid mask at night). Having a better understanding of subtypes of DED, which are in part specific to relevant comorbidities, can be translated into more tailored treatment interventions.

## Figures and Tables

**Table 1:** Demographic and clinical characteristics of the study sample by DM subgroup.

	Controls (n = 272)	DM − C (n = 53)	DM + C (n = 41)	*p*-Value
Demographics, % (n)				
Age, years, mean ± SD	59.0 ± 6.5	58.2 ± 6.4	61.7 ± 5.2 ^b^	0.02
Sex, male	88% (238)	91% (48)	100% (41)^a^	0.05
Race, White	40% (109)	30% (16)	41% (17)	0.20
Ethnicity, Hispanic	23% (62)	26% (14)	34% (14)	0.27
Clinical data, mean ± SD (n)				
HbA1C, %	6.1 ± 1.7 (19)	7.0 ± 1.3 (52) ^a^	8.0 ± 1.9 (40) ^b^	<0.001
Weight, pounds	194.3 ± 41.9 (270)	221.5 ± 45.7 (52) ^a^	226.9 ± 34.2 (41) ^a^	<0.001
BMI (kg/m^2^)	28.6 ± 5.8 (270)	32.7 ± 5.9 (52) ^a^	32.8 ± 3.6 (41) ^a^	<0.001
Comorbidities, % (n)				
Smoking (current)	44% (119)	40% (21)	37% (15)	0.62
Hypertension	61% (166)	92% (49) ^a^	85% (35) ^a^	<0.001
Hypercholesterolemia	47% (128)	83% (44) ^a^	85% (35) ^a^	<0.001
PTSD	22% (60)	25% (13)	29% (12)	0.58
Depression	70% (190)	62% (33)	61% (25)	0.34
Arthritis	51% (139)	49% (26)	56% (23)	0.78
Sleep apnea	23% (62)	36% (19)	24% (10)	0.13
BPH	11% (31)	21% (11)	24% (10) ^a^	0.03
Rosacea	3% (7)	0% (0)	0% (0)	0.29
Hepatitis C	15% (40)	11% (6)	7% (3)	0.39
Devices and medications, % (n)				
CPAP	5% (13)	6% (3)	10% (4)	0.43
NSAIDs	40% (108)	47% (25)	20% (8) ^b^	0.02
ASA	33% (91)	66% (35) ^a^	59% (24) ^a^	<0.001
Fish oil	7% (19)	15% (8)	7% (3)	0.14
Multivitamins	48% (130)	53% (28)	63% (26)	0.16
Beta blockers	17% (46)	25% (13)	32% (13)	0.05
Statins	35% (96)	79% (42) ^a^	83% (34) ^a^	<0.001
Antidepressants	60% (162)	47% (25)	46% (19)	0.09
Anxiolytics	57% (154)	49% (26)	49% (20)	0.44
Analgesics	69% (187)	74% (39)	61% (25)	0.42
Antihistamines	23% (63)	25% (13)	20% (8)	0.84
Alpha-2-delta ligands	31% (85)	36% (19)	34% (14)	0.78
Venlafaxine	1% (3)	0% (0)	0% (0)	0.59
Sildenafil	33% (90)	38% (20)	34% (14)	0.81

DM: diabetes mellitus; DM − C: diabetes mellitus without end-organ complications; DM + C: diabetes mellitus with end-organ complications; SD: standard deviation; HbA1C: most recent glycosylated hemoglobin; BMI: body mass index; kg: kilograms; m: meters; PTSD: post-traumatic stress disorder, BPH: benign prostatic hyperplasia; CPAP: continuous positive airway pressure; NSAID: non-steroidal anti-inflammatory drugs; ASA: acetylsalicylic acid. ^a^ Statistically significant difference compared to controls. ^b^ Statistically significant difference compared to controls and DM − C.

**Table 2:** Symptoms and signs in individuals grouped by DM subtype.

DE Metric [Range] (n)	Controls (n = 272)	DM − C (n = 53)	DM + C (n = 41)	*p*-Value
DED and ocular pain symptoms quantified based on questionnaires [range] (n), mean ± SD				
DEQ-5 [0–22] (366)	12.21 ± 4.55	11.83 ± 5.09	11.56 ± 4.75	0.65
OSDI [0–100] (366)	42.08 ± 24.46	38.88 ± 25.13	23.63 ± 16.20 ^b^	<0.001
NRS [0–10] (366)	4.89 ± 3.17	4.34 ± 2.72	3.54 ± 2.68 ^a^	0.02
NPSI-Eye [0–40] (366)	13.22 ± 10.73	11.06 ± 10.51	7.59 ± 7.97 ^a^	0.004
Corneal sensation assessed using Belmonte aesthesiometer (right eye) [range] (n), mean ± SD				
Detection threshold, ml/min [12.5–240] (360)	79.60 ± 38.44	85.29 ± 38.42	87.32 ± 33.17	0.34
Pain threshold, ml/min [25–410] (346)	233.75 ± 118.30	225.30 ± 116.20	248.51 ± 120.06	0.66
Tear film osmolarity (value from more severely affected eye) [range] (n), mean ± SD				
Osmolarity, mOsmol/L [277–380] (342)	305.43 ± 15.58	308.53 ± 19.00	304.38 ± 12.77	0.38
Osmolarity difference between eyes, mOsmol/L [0–86] (330)	9.97 ± 12.12	11.98 ± 15.68	8.94 ± 6.14	0.48
Ocular surface evaluation (value from more severely affected eye) [range] (n), mean ± SD				
Floppy eyelids, upper and lower [0–2] (355)	0.69 ± 0.64	0.73 ± 0.72	1.08 ± 0.77 ^b^	0.004
MG plugging [0–3] (365)	1.71 ± 0.91	1.75 ± 0.83	1.78 ± 0.91	0.87
Eyelid vascularity [0–3] (365)	0.52 ± 0.71	0.26 ± 0.56 ^a^	0.59 ± 0.67 ^c^	0.03
Conjunctivochalasis sum [0–3] (351)	1.51 ± 0.98	1.20 ± 1.03 ^a^	1.81 ± 1.00 ^c^	0.02
TBUT, seconds [0–15] (362)	9.63 ± 4.91	8.98 ± 4.95	9.84 ± 4.85	0.63
CFS [0–15] (364)	1.85 ± 2.42	2.15 ± 2.23	2.29 ± 2.09	0.43
Schirmer’s test, mm wetting [0–35] (362)	12.97 ± 7.53	13.72 ± 7.60	11.73 ± 5.76	0.43
Meibum quality [0–4] (358)	1.85 ± 1.21	1.79 ± 1.31	2.05 ± 1.28	0.57
MG dropout [0–4] (365)	1.41 ± 0.95	1.43 ± 1.02	1.76 ± 1.04	0.10

DE: dry eye; DM: diabetes mellitus; DM − C: diabetes mellitus without end-organ complications; DM + C: diabetes mellitus with end-organ complications; SD: standard deviation; DEQ-5: Dry Eye Questionnaire-5; OSDI: Ocular Surface Disease Index; NRS: numerical rating scale; NPSI-Eye: Neuropathic Pain Symptoms Inventory Modified for the Eye; MG: meibomian gland; TBUT: tear break-up time; CFS: corneal fluorescein staining. ^a^ Statistically significant difference compared to controls. ^b^ Statistically significant difference compared to controls and DM − C. ^c^ Statistically significant difference compared to DM − C. Data are provided as available and may not sum to the sample total.

**Table 3:** Forward stepwise linear regression model considering DM status, ocular symptoms, and anatomy.

			Unstandardized Coefficients	Standardized Coefficients		Coefficient of Determination	
Dependent Variable	Model	Predictor	B	SE	β	*p*-Value	R^2^
OSDI	1	DM 0–2	−7.95	1.84	−0.22	<0.001	0.05
	2	DM 0–2	−6.19	1.97	−0.17	0.002	0.06
		Statins	−6.33	2.67	−0.13	0.018	
	3	DM 0–2	−5.89	1.97	−0.16	0.003	0.07
		Statins	−6.14	1.97	−0.13	0.022	
		Age	−0.391	2.66	−0.10	0.043	
Floppy eyelids, upper and lower	1	Age	0.03	0.01	0.24	<0.001	0.06
	2	Age	0.03	0.01	0.24	<0.001	0.08
		DM 0–2	0.14	0.05	0.14	0.01	
	3	Age	0.03	0.01	0.24	<0.001	0.09
		DM 0–2	0.12	0.05	0.11	0.031	
		BMI	0.01	<0.01	0.11	0.05	

B: beta coefficient; SE: standard error; β: standardized coefficient; R^2^: total variability explained by the model; OSDI: Ocular Surface Disease Index; DM: diabetes mellitus status from 0 to 2, where 0 = no DM, 1 = DM without end-organ complications, and 2 = DM with end-organ complications.

## Data Availability

The data presented in this study are available from the corresponding author upon reasonable request.
